# Processing of Metals and Metalloids by *Actinobacteria*: Cell Resistance Mechanisms and Synthesis of Metal(loid)-Based Nanostructures

**DOI:** 10.3390/microorganisms8122027

**Published:** 2020-12-18

**Authors:** Alessandro Presentato, Elena Piacenza, Raymond J. Turner, Davide Zannoni, Martina Cappelletti

**Affiliations:** 1 Department of Biological, Chemical and Pharmaceutical Sciences and Technologies (STEBICEF), University of Palermo, 90128 Palermo, Italy; elena.piacenza91@gmail.com; 2 Department of Biological Sciences, Calgary University, Calgary, AB T2N 1N4, Canada; turnerr@ucalgary.ca; 3 Department of Pharmacy and Biotechnology (FaBiT), University of Bologna, 40126 Bologna, Italy; davide.zannoni@unibo.it (D.Z.); martina.cappelletti2@unibo.it (M.C.)

**Keywords:** metal resistance mechanisms, actinobacteria, metal stress response, metal-based nanostructures, biogenic nanoscale materials

## Abstract

Metal(loid)s have a dual biological role as micronutrients and stress agents. A few geochemical and natural processes can cause their release in the environment, although most metal-contaminated sites derive from anthropogenic activities. Actinobacteria include high GC bacteria that inhabit a wide range of terrestrial and aquatic ecological niches, where they play essential roles in recycling or transforming organic and inorganic substances. The metal(loid) tolerance and/or resistance of several members of this phylum rely on mechanisms such as biosorption and extracellular sequestration by siderophores and extracellular polymeric substances (EPS), bioaccumulation, biotransformation, and metal efflux processes, which overall contribute to maintaining metal homeostasis. Considering the bioprocessing potential of metal(loid)s by Actinobacteria, the development of bioremediation strategies to reclaim metal-contaminated environments has gained scientific and economic interests. Moreover, the ability of Actinobacteria to produce nanoscale materials with intriguing physical-chemical and biological properties emphasizes the technological value of these biotic approaches. Given these premises, this review summarizes the strategies used by Actinobacteria to cope with metal(loid) toxicity and their undoubted role in bioremediation and bionanotechnology fields.

## 1. Introduction

Metals [e.g., silver (Ag), aluminum (Al), cadmium (Cd), cobalt (Co), chromium (Cr), cesium (Cs), copper (Cu), mercury (Hg), iron (Fe), magnesium (Mg), manganese (Mn), molybdenum (Mo), nickel (Ni), lead (Pb), strontium (Sr), zinc (Zn), uranium (U)] and metalloids [i.e., arsenic (As), selenium (Se), tellurium (Te)], although naturally occurring throughout the Earth’s crust, are not generally present in soluble forms, but suspended as colloids, or in association with organic matters, minerals, and rocks [1]. The release of metals and metalloids (henceforth indicated as metal(loid)s) into the environment derives from geochemical alterations, the type and age of the rock material, as well as natural events, such as volcanic eruptions, atmospheric deposition, leaching of the Earth, earthquakes, typhoons, and climate changes. Some metal(loid)s (e.g., Cu, Co, Fe, Mn, Se, Zn) are essential trace micronutrients needed to support several biological functions in all the domains of life, acting as cofactors featuring important structural and catalytic properties in enzymes and proteins, electron carriers, and regulators of the cellular osmotic pressure [2]. As opposed to this scenario, the presence of “*non-physiological*” levels of metal(loid)s into the environment has nowadays gained growing concern worldwide, and the reasons behind this phenomenon must be sought elsewhere. Anthropogenic activities (e.g., alloy production, use of pesticides, leather tanning, battery production, and electroplating industries) inexorably determined the buildup of wastes containing metal(loid)s during the 20th century. Moreover, metal(loid) anthropogenic source is more bioavailable and (bio)reactive as compared to those normally found in nature, being this aspect strictly correlated to the enhanced metal(loid) mobility and accumulation at different levels of the trophic chain [3]. Indeed, the reduction or destruction of these substances is a challenging task, making the excess of metal(loid)-containing compounds in the environment a persisting long-term problem [4]. In the case of metal(loid)s, they can cause severe environmental and human health issues even at low concentrations, exerting mutagenic and carcinogenic effects and impairing, among others, the central and peripheral nervous system or the circulatory one [5]. The toxic effects of metal(loid)s depend on the chemical element itself and the load at which it is encountered, as well as a variety of physical-chemical (e.g., temperature, pH, redox-potential, presence/absence of chelating compounds) and biological parameters (e.g., the metal(loid)-lipid solubility, physiological state of the organism, presence/absence of metal interacting proteins, organism adaptation phenomena), including also the way all these factors interplay with each other [6]. Furthermore, the overexposure to metal(loid)s can both compromise soil and water micro-flora fitness, severely worsening the chances of recovering environments contaminated by new xenobiotics [7,8], and contribute to the emergence, maintenance, and transmission of antimicrobial-resistant pathogens [9,10,11]. Among possible solutions for metal(loid) removal or recovery, chemical precipitation, ultrafiltration, ion-exchange, reverse osmosis, and electro-dialysis are procedures that generate toxic wastes and are economically disadvantageous, mostly considering the large-scale application necessary to face the diffuse contamination of metal(loid)s [12,13]. Conversely, bioremediation strategies represent promising eco-friendly, economically feasible, efficient, and pollutant-selective approaches, involving the use of metal(loid) tolerant and/or resistant microorganisms [14], which have evolved specific metabolic processes and adaptation mechanism(s) associated with their survival under different stress conditions [15]. In particular, the survival and development of bacteria in extreme and harsh environments (e.g., in the presence of toxic metals) is allowed by the evolution of inducible mechanisms associated with specific genetic traits (i.e., referred to as resistance) and/or adaptation ones related to intrinsic biochemical features (i.e., referred to as tolerance) [15]. These metabolic activities can be exploited to lower metal(loid) concentration to acceptable and safe levels in order to restore contaminated ecological niches and metal transformation for their recovery (e.g., metal-based nanostructure bioproduction) [16].

Among bacteria that are frequently recovered from toxic metal(loid) and, in turn, utilized for bioremediation strategies, members of Actinobacteria phylum have acquired extensive research attention. Actinobacteria are distributed among 221 phenotypically and morphologically different genera that are highly ubiquitous on Earth and inhabit diverse and extreme environments (e.g., water, soil, plant surface, arctic, and deserted areas) [17]. This bacterial group includes microorganisms exhibiting highly variable physiological and metabolic properties as a reflection of their wide genomic heterogeneity [17]. Among Actinobacteria, members of *Streptomyces* genus and the CMNR group (*Corynebacterium*, *Mycobacterium*, *Nocardia*, and *Rhodococcus*) possess large genomes and multiple linear and circular megaplasmids, which contribute to their extraordinary metabolic capabilities and toxic metal resistance [18,19,20]. One interesting aspect of combining bioremediation with metal recovery strategies involves the utilization of microbes to produce nanostructures (NSs) containing metal(loid)s, which are typically more toxic in bulk. Metal(loid) nanomaterials (MeNMs) play a central role in nanotechnology, the field of science dealing with materials that possess at least one dimension in the nanorange (10^−9^ m), where they hold physical-chemical characteristics (high surface-to-volume ratio, large surface energy, and spatial confinement) from which a plethora of intriguing properties—e.g., size-dependent, optical, catalytic, electronic, mechanical—arise as compared to the corresponding bulk materials. This aspect has enabled the extensive use of metal(loid) nanomaterials (MeNMs) at a multidisciplinary level in various application fields [21,22].

Given these premises, this review will overview the mechanisms of interaction between some model strains of Actinobacteria phylum and metal(loid)s in terms of (i) resistance strategies adopted to counteract metal(loid) toxicity, (ii) transformation of metal(loid) ion/oxyanions into less toxic and bioavailable forms, (iii) synthesis of biogenic MeNMs with different physical-chemical features (e.g., size, morphology, and properties) and relevant biotechnological applications.

## 2. Mechanism(s) of Metal Tolerance and Resistance in Actinobacteria

**Figure 1:**
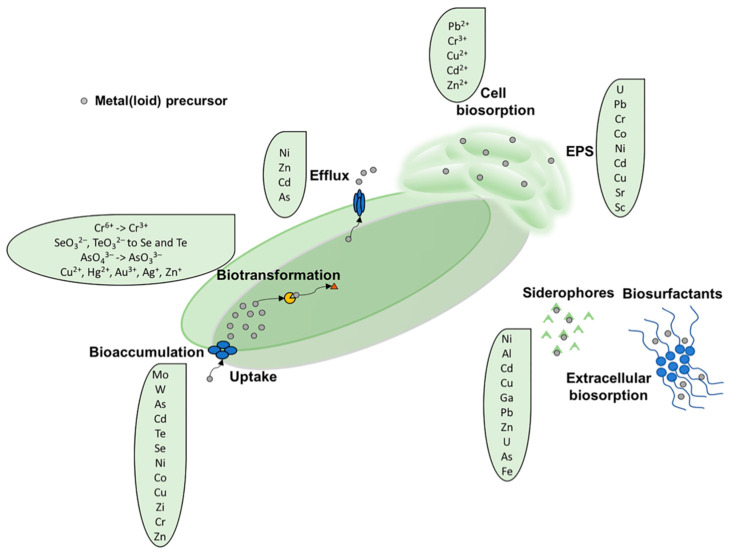
Schematic representation of biotic mechanisms (i.e., biosorption and extracellular sequestration by siderophores, biosurfactants, and extracellular polymeric substances (EPS), bioaccumulation, biotransformation, and metal efflux processes) of interaction between Actinobacteria and metal(loid)s.

Various members of Actinobacteria phylum hold a positive and long-standing reputation for environmental biotechnology applications, as they (i) can utilize inexpensive and/or toxic compounds as carbon and energy sources, (ii) feature an environmental resilience, persistence, and robustness– which derives from a versatile catabolic and anabolic potential, (iii) usually are not pathogens, and (iv) generally display a very good basal resistance to metals. All these aspects make Actinobacteria excellent candidates for the bioremediation of metal(loid)-polluted environments [23,24,25]. The main actinobacterial tolerance/resistance strategies to metal(loid) toxicity is associated with biosorption, bioaccumulation, and biotransformation processes [26], which are visually summarized in Figure 1 and described in the paragraphs below.

### 2.1. Biosorption

Biosorption represents a microbial tolerance mechanism that allows the lowering of the local metal(loid) concentration. This process occurs through electrostatic interactions between metal(loid) ions and functional groups present on microbial surfaces such as proteins, carbohydrates, cell wall polymers, extracellular polymeric substance (EPS), and metal chelating factors (e.g., siderophores and metalloproteins) [27]. Biosorption is recognized, from an industrial perspective, as a highly efficient, economically, and ecologically sustainable process since it avoids the formation of hazardous wastes in removing metal(loid)s, radionuclides, and toxic organic xenobiotics from polluted environments. From a physical-chemical perspective, microorganisms are generally considered good biosorbents due to their small size and high surface-to-volume ratio, thus offering a large surface area for their interaction with metal(loid)s to successfully occur [28,29,30,31].

Depending on the metal(loid) speciation found in a specific ecological niche [3] and its physical-chemical factors, diverse members of Actinobacteria phylum have been described to carry out metal(loid) sorption strategies [32]. The affinity, yield, and specificity of this bioprocess rely on the cell wall and membrane surface structural chemistry—i.e., charge and orientation of the metal(loid)-binding groups [33]. Indeed, the actinobacterial cell surface has a complex macromolecular composition (lipids, peptidoglycan, teichoic acid, lipo- and surface-proteins, enzymes and mycolic acids) that overall expose diverse reactive functional groups (e.g., hydroxyl [OH^−^], carbonyl [CO], carboxyl [COO^−^], phosphate [PO_4_^3−^], sulfate [SO_4_^2−^], and amino [NH_2_^+^/NH_3_^+^]), which can mediate the sorption and deposition of metal(loid)s on the bacterial surface [34,35,36]. For instance, *S. zinciresistens* exhibited a bald—no spore-forming—phenotype, which showed a short and thin mycelium upon cell exposure to Zn^2+^ and Cd^2+^, likely due to the interaction occurring between OH^−^, CO, and NH surface moieties and these cations, resulting in dense granules visible on the cell surface by scanning electron microscopy (SEM) [37]. The availability of proton binding sites, as a function of pH and salt level, will determine a diverse net charge of the actinobacterial cell surface, widening the possibility of eliciting different sorption phenomena. This impacts the general pKa, isoelectric point, and the number of active moieties of the biosorbent, as highlighted in work carried out by Plette and colleagues (1995) using *Rhodococcus erythropolis* A177 [34]. At pH values lower than the isoelectric point of the biosorbent, the biosorbent will feature a positive net charge due to its association to hydronium (H_3_O^+^) ions, therefore repulsing metal(loid) cations. The opposite effect (i.e., negative net charge) is expected at pH values higher than the biosorbent isoelectric point, leading to the cation adsorption. In this context, the biosorption capacity of Al^3+^ by *R. opacus* strain was enhanced at pH values around 5, as the general isoelectric point of this biosorbent was 3.26, as well as in the case of other metal(loid)s (e.g., Pb^2+^, Cr^3+^, Cu^2+^, Cd^2+^, and Zn^2+^) [38,39,40]. Moreover, proton exchange with metal(loid)s will be favored in the case of pH values above the pKa of the binding moieties characterizing the biosorbent [41]. Fourier-transform infrared (FTIR) spectroscopy analyses performed on *Streptomyces* VITSVK5, VITSVK9, and *S. werraensis* LD22 strains exposed to Pb^2+^, Cd^2+^, and Cr^3+^ revealed that ion-exchange takes place between metals and hydrogens of OH^−^, COO^−^, and NH_2_^+^ groups [42,43,44]. The importance of these charged moieties for actinobacterial biosorption was further proved by their chemical modifications, i.e., through methylation of NH_2_^+^/NH_3_^+^ groups or esterification of both OH^−^ and COO^−^ ones that dramatically impaired the sorption of Cu^2+^ as compared to untreated biomass [45].

If the pH, the type of metal(loid), and the nature of the biosorbent material represent important factors that must be accounted for the optimization of biosorption processes, in natural settings, the contemporary presence of a variety of metal(loid)s may lead to their competition for the ligand sites present on the surface of actinobacterial cells, compromising sorption bioprocesses. This is the case of Ca^2+^ that is the main competitor for metal sorption in contaminated soil matrices [46,47,48], yet *R. erythropolis* A177 showed good sorption capacity towards Cd^2+^, even in the presence of Ca^2+^ ions. Upon exposure of *Rhodococcus* cells to Ca^2+^, the cell wall drastically changed because of breakings of the cross-links between different chemical surface moieties and alteration in the structure of the peptidoglycan layer, increasing the number of active binding sites for Cd^2+^ [49]. On the other hand, it has been reported that the cell amount in a specific contaminated site influences the single-cell sorption capacity, likely due to cross-links between functional groups [39,41,50].

#### 2.1.1. Extracellular Sequestration by Siderophores

The scarce aqueous solubility and bioavailability of Fe and its importance for several biological functions led microorganisms to evolve specific mechanisms of Fe-uptake and Fe-trapping, mostly represented by siderophore biosynthesis [51,52]. Siderophores are low-molecular-weight (200–2000 Da) and diffusible molecules featuring a high affinity and selectivity for the insoluble ferric (Fe^3+^) as compared to ferrous (Fe^2+^) cations, which guarantees a low level of competition with other bivalent yet essential metal cations(e.g., Zn^2+^, Cu^2+^, Ni^2+^, and Mn^2+^) [53]. Thus, siderophores’ biosynthesis and secretion usually depend on Fe^3+^ environmental concentration [52,53,54], as they act as Fe-chelators, creating extracellular iron-siderophore complexes from which Fe^3+^ is taken up by bacterial cells through redox processes [53,55]. After the first study reporting the production of the siderophores arthrobactin and mycobactin by *Arthrobacter terregens* and *Mycobacterium johnei* in the 1950s [53], actinobacterial members have been described to be able to synthesize siderophores, which, depending on their chemical structure, are indicated as phenolates, catecholates, carboxylates, hydroxamates, or mixed types [56,57,58] (Table 1).

Among these, hydroxamates (e.g., desferrioxamines G1, B, and E, rhodotorulic acid, coelichelin) appear to be the most common [54,74,75]. *Streptomyces* spp. were also found to produce catecholate siderophores, such as enterobactin and myxochelin, although they are typical of Gram-negative bacterial strains [54]. The production of structurally diverse siderophores seems to follow either the desferrioxamine or the multiple nonribosomal peptide synthetase (NRPS) biosynthetic pathways, which are upregulated under Fe-deficiency [54]. Although it is not obvious to understand the advantage of Actinobacteria and, specifically, of *Streptomyces* spp., to biosynthesize diverse siderophores, this phenomenon may depend on the need of these bacterial strains to face competitive interactions with other microorganisms [54]. Recent studies also demonstrated the ability of actinobacterial siderophores to bind several toxic metal(loid)s other than Fe^3+^, contributing to their bioavailability reduction and, therefore, enhancing the bacterial survival in contaminated environments [52,65,76]. In this regard, hydroxamates produced by *Streptomyces* spp. can chelate Ni, Al, Cd, Cu, gallium (Ga), Pb, Zn, U, and As, although the binding strength of these metal(loid)s is lower as compared to Fe [63,65,69,71,76]. Once metal(loid)s form a complex with siderophores, they can either be assimilated or sequestered in the extracellular environment, the latter alleviating the cell stress deriving from toxic metal(loid)s [63,69,72]. Particularly, coelichelin, desferrioxamines, and hydroxamates produced by *S. acidiscabies* E13 and *S. tendae* F4 showed good binding selectivity and, therefore, toxicity protection towards Ni^2+^ *Leifsonia* spp. and Cd^2+^ [63,64,65]. In this regard, the low amount of available Fe^3+^ in the culture media, and its competition with other metals, likely stimulated an active biosynthesis of siderophores by these bacteria to obtain enough Fe-content for their survival [64,65]. Catecholates and phenolates were also found responsible for counteracting Cd^2+^ toxicity in a wide number of *Streptomyces* spp., suggesting the presence of regulatory systems for siderophores’ synthesis in these microorganisms [71]. Siderophores were described to be involved in the post-efflux chelation of Cd and Zn also in *Methylobacterium* and *Frigobacterium* spp. [66,77], and in the binding and chelating of As^3+^ cations in *A. oxydans* ATW2 and ATW3, *Kokuria rosea* ATW4, *R. erythropolis* ATW1 and S43 under Fe-deficiency [72]. Particularly, Fe- or As-siderophore complexes extracted from *A. oxydans* ATW3 and *R. erythropolis* ATW1 showed distinct physical-chemical characteristics, indicating the occurrence, in Actinobacteria, of multiple co-existing chelators with different metal(loid) selectivity [16,72].

#### 2.1.2. Extracellular Sequestration Mediated by Extracellular Polymeric Substance (EPS)

A common feature of bacteria is the production and release of biological exudates, such as polysaccharides and their derivatives, lipids, secondary metabolites, proteins, and nucleic acids, which are generally referred to as EPS [78,79]. These extracellular biomolecules constitute a complex hydrogel matrix that confers fluid and elastic properties to microorganisms, allowing them to adapt to environmental changes, particularly when growing as biofilm [80]. EPS forms a protective layer surrounding bacterial cells, whose physical-chemical structure guarantees to microorganisms an efficient adhesion to surfaces, the accumulation and dissolution of nutrients, and the protection towards desiccation, predation, as well as toxic and exogenous substances, such as metal(loid)s, antimicrobials, and reactive oxygen species (ROS) present in the environment [52,78]. EPS features a great ability to bind and immobilize metal(loid) ions from the extracellular environment, avoiding their entry within bacterial cells and, hence, playing a fundamental role as biosorbents for these substances [81,82,83]. The biosorption capability of EPS is selective, and it depends on (i) its structure and functional groups, (ii) environmental parameters (i.e., pH, temperature, and carbon source availability), and (iii) the initial concentration, size, and bond energy of the metal(loid) ions in the extracellular environment [81,83,84,85]. Overall, the high hydrophilicity, chemical reactivity, and stability of polysaccharides make them good biosorbents, as they display several chemical reactive groups [e.g., COO^−^, CO, NH_3_^+^, OH^−^, PO_4_^3−^, and acetamido (AcNH^+^)] that can dynamically interact and sequester metal(loid) ions [78,86]. EPS metal(loid) biosorption occurs through electrostatic interactions between the charged biomolecules and metal(loid)s, forming stable metal-EPS complexes [27,81,83,87,88]. The biosorption potential of EPS is correlated with the number of functional groups present within this matrix, enhancing the metal(loid) binding to bacterial cell membranes [89,90]. Once EPS immobilizes metal(loid) ions, they can be sequestered and transformed by enzymes and proteins through ion exchange, complexation, and precipitation [27,52].

Several members of the Actinobacteria phylum were demonstrated to produce EPS in response to metal stress, representing the first defense mechanism against the toxicity of these inorganic compounds (Table 2). Within this phylum, EPS biosynthesis is influenced by the bacterial species, the growth phase, the availability of nutrients, and the selective pressure exerted by toxic compounds [84,88].

*Amycolatopsis* sp. AB0 produced EPS mainly constituted by polysaccharides containing ca. 20 repeating glucose units that could bind Cu^2+^ [92]. Upon addition of Cu^2+^ and Pb^2+^, cells of *K. rizophila* (former *Micrococcus luteus*) DE2008 enhanced the secretion of EPS that was characterized by a larger amount of carbohydrate and uronic acid moieties as compared to the EPS produced in the absence of the metal challenge, which determined an improvement in metal immobilization efficiency [94]. The role of EPS in metal biosorption by the DE2008 strain was suggested by the formation of Cu and Pb deposits visible in the extracellular matrix [93,94]. Stable precipitates of Pb^2+^ were also detected on the cell surface of *Frankia* sp. strain EAN1pec, in which a specific Pb-binding mechanism was demonstrated to occur. The involvement of EPS, as well as cell wall lipids, in the adsorption of Pb^2+^, was suggested by proteomic analyses, which highlighted peculiar surface and lipid–protein expression patterns in EAN1pec cells exposed to Pb^2+^ [98].

The EPS biosorption behavior towards different metal cations and the influence of metal concentration, pH, and ionic strength on biosorption efficiency was assessed using cultures of *Arthrobacter* ps-5 [81], *R. opacus* 89 UMCS, and *R. rhodochrous* 202 DSM [86]. These actinobacterial strains showed a biosorption preference towards Pb cations followed by Cu and Cr in the case of *Arthrobacter* ps-5 [81] and Co, Cr, Cd, and Ni for *Rhodococcus* spp. strains [86]. Particularly, the adsorption equilibrium was reached within 30 min of incubation for all the metals tested for both 89 UMCS and 202 DSM strains, being the highest Pb biosorption rate observed with the latter strain (ca. 200 mg/g Pb^2+^ in 5 min) [86]. The EPS secreted by *Arthrobacter* ps-5 contained polysaccharides composed of glucose and galactose units, whose amount strongly influenced its metal biosorption ability, as a high number of functional groups acting as binding sites for metal cations were available [81]. Specifically, functional groups containing oxygen [i.e., OH^−^, COO^−^, CO, and ether (COC)] were involved in the complexation of metal cations in *Arthrobacter* ps-5 and *Rhodococcus* spp. [81,86], due to the oxygen tendency to reduce the electron cloud density of these chemical groups [101]. However, reactive NH_3_^+^, methyl (CH_3_), methylene (CH_2_), primary (NH) and secondary (NH_2_) amine groups were also found to be partially responsible for EPS biosorption of Ni^2+^, Pb^2+^, Co^2+^, Cd^2+^, and Cr^4+^ in the case of rhodococci [86]. Moreover, the pH must be accounted for the biosorption process, as H_3_O^+^ ions present in an acid (pH = 2–3) solution could compete with Pb, Cu, Co, and Cr cations for the same EPS functional groups [102,103], while at pH > 6 these metals can precipitate in the medium forming metal hydroxides [104], impairing their immobilization within the bacterial EPS. Thus, a pH value of 5 was the optimal condition for Pb, Cu, Co, and Cr biosorption in *Arthrobacter* ps-5 cultures [81] and *Rhodoccus* spp. [86], while the latter better immobilized Cd^2+^ cations when the pH was fixed at 6.5, as, at values greater than 8, Cd(OH) complexes dominate [86]. A similar conclusion can be drawn for the greatest Ni^2+^ EPS-mediated adsorption in the case of rhodococci, which occurred at pH between 3 and 4, as higher values led to the formation of Ni(OH)_2_ and Ni(OH)_3_ species, whose interaction with EPS’ functional groups are less favored [86]. A competition for the EPS binding sites can also arise between metal cations and metal salts (e.g., KCl and CaCl_2_) generally present in the growth medium, as the positive charges of the latter can electrostatically interact with the actinobacterial extracellular matrix [81]. In this regard, a decrease in the EPS biosorption ability was detected when metal salt concentration was increased in cultures of *Arthrobacter* ps-5, being Ca^2+^ more effective in competing with Pb, Cd, and Cr [81].

In some Actinobacteria members, EPS-mediated biosorption seemed to be also involved in the detoxification of metal radionuclides, such as strontium (Sr) and cesium (Cs) [87,88,100]. Indeed, *Nocardiopsis* sp. 13H could adsorb both Sr^+^ and Cs^+^ species through the production of EPS, which featured layers that were loosely (outer and prone to dissolution) and tightly (inner) bound to bacterial cells [87,100]. Compositional analysis of EPS revealed that the carbohydrate content largely increased in both layers, consequently to Sr or Cs exposure, followed by proteins, nucleic acids, and unidentified compounds. Moreover, Sr^+^ and Cs^+^ were only found in the inner layer of EPS, which featured a large number of OH^−^, PO_4_^3−^, NH_2_ and thiol (SH) groups that probably have a significant role in the biosorption of these metal radionuclides [87,100]. Similarly, EPS produced by *Streptomyces* sp. CuOff24 showed a good proficiency as a biosorbent for Sr^+^, being arabinose, galactose, mannose, glucose, and uronic acid the major components identified along with NH, NH_2_, AcNH^+^, and SH reactive moieties [88].

### 2.2. Bioaccumulation

A further strategy that microorganisms can exploit to detoxify the surrounding environment from metal(loid)s is based on bioaccumulation phenomena, which imply the active transport of metals within the cells through specific uptake systems [3,105,106]. Bioaccumulation occurs through slow metabolic-dependent and energy-demanding processes, which generally follow the rapid metal(loid) biosorption by bacteria, resulting in a net decrease of the metal(loid) concentration in the extracellular environment. Thus, bioaccumulation can be performed only by living cells that feature specific genetic and biochemical assets involved in metal transport and can undergo physiological adaptations in response to the presence of metal(loid)s [107]. Moreover, it has been reported that the temperature can affect metal(loid) bioaccumulation as if, on one hand, a temperature increase is expected to enhance the rate of chemical reactions; on the other hand, it can influence the fluidity of biological membranes, compromising the respiratory chain kinetics and permeability of microbial cell, which are crucial for the bacterial survival [108]. Once inside the cells, some metal(loid)s (e.g., As, Cd, Te, Se) can be used as terminal electron acceptors and/or cofactors in metalloproteins and enzymes [109,110,111]. They can also be transformed through either redox processes or alkylation reactions [3], where such changes in speciation can alter metal(loid) mobility and toxicity. The internalization of metal(loid)s in the bacterial cytoplasm can be mediated by (i) ion channels or carriers exploiting a concentration gradient and (ii) active transporters dependent on either ATP consumption or the electrochemical gradient as an energy source. This aspect is particularly important for those bacteria holding enzymatic activities (e.g., nitrogenases and oxidoreductases) that use molybdoproteins as cofactors, making it imperative for these microorganisms to acquire Mo (as the oxyanion MoO_4_^2−^), which generally occurs through either the high-affinity ATP-binging cassette (ABC) transporter or low-affinity carrier systems [112]. Non-essential and toxic metal(loid)s can be internalized through promiscuous transport systems typically involved in the uptake of the essential ones, as in the case of the molybdate ABC transporter that does not discriminate tungsten (W) in its oxyanion from (i.e., WO_4_^2−^), which can therefore be bioaccumulated. In regard to the oxyanions, those containing metalloid elements, such as arsenate (AsO_4_^3−^), arsenite (AsO_3_^3−^), tellurate (TeO_4_^2−^), tellurite (TeO_3_^2−^), selenate (SeO_4_^2−^), and selenite (SeO_3_^2−^), are extremely toxic for many living organisms; for instance, TeO_3_^2−^ exert its toxicity at a concentration as low as 1 µg mL^−1^ towards both prokaryotic and eukaryotic organisms [113]. Moreover, although Se represents an essential micronutrient for living systems [114], the overexposure to Se-compounds can cause severe health issues, while any biological function has not been reported for those containing As and Te [115]. Due to anthropogenic activities, these oxyanions can now be found as pollutants at critical environmental concentrations, whose toxicity relies on their high reactivity, mobility—through soil and water streams—and bioavailability thanks to their association with oxygen [116]. Despite the oxyanions’ toxicity, bacteria can take up either SeO_4_^2−^ or SeO_3_^2−^ using transporters and/or permeases involved in sulfate and sulfite uptake [117,118,119] and using polyol ABC transporters [120]. Further, the phosphate transporter has been proposed to be involved in the internalization of TeO_3_^2−^ and AsO_4_^3−^, since these oxyanions are strong competitors of PO_4_^3−^ groups [121,122,123,124,125], as well as the acetate permease (ActP) monocarboxylate transporter [126].

Other than oxyanions, bacterial strains have evolved specific systems of bioaccumulation to assure a proper supply of metal cations and detoxification bioprocesses. In this context, Abbas and Edwards (1990), alongside Amoroso and colleagues (1998), isolated diverse *Streptomycetes* that showed significant resistance levels against Hg^2+^, Co^2+^, Cd^2+^, Cu^2+^, Zn^2+^, Cr^6+^, Ni^2+^, and Mn^2+^ [127,128,129]. Some of these cations were taken up by bacterial cells exploiting the transport system of Mg^2+^, which represents a strong competitor for the uptake of transition metals [129]. Transporters that can be used for Ni^2+^ internalization are high-affinity permeases that are characterized by low transport capacity [130,131,132]. In the case of *M. tuberculosis* and *Streptomyces* spp., it has been reported that they can utilize these permeases to support the activity of Ni-dependent ureases [133] and superoxide dismutases (Sod), respectively. Particularly, Ni plays a crucial role in *S. coelicolor*, regulating *sod* gene expression, post-translational modifications, and Sod enzymatic activity [134]. Moreover, the Ni-responsive transcriptional regulator Nur, a homolog of the iron one (Fur), was identified as responsible for Ni uptake in *S. coelicolor*, binding the promoter region of *nikA* of the putative Ni transporter gene cluster (*nikABCDE*) [135]. On the same line, Lu and colleagues (2014) deciphered, in *S. coelicolor* A3(2), the molecular function of a hypothetical protein that binds four Ni^2+^ at its surface. The gene coding for this protein is only upregulated by the presence of Ni^2+^ as compared to other cations, such as Cu^2+^, Co^2+^, and Mn^2+^ [136]. This evidence underlines how the intracellular nickel homeostasis and antioxidative response are finely regulated in *S. coelicolor*. On the other hand, *R. rhodochrous* strain J1 used the Nh1F transporter to take up physiological amounts of Co^2+^ to sustain the nitrile hydratase activity, which exploits non-corrin Co as a cofactor [137]. In line with this, Pogorelova and colleagues (1996) described the importance of Co^2+^ for the nitrile utilization by *R. rhodochrous* strain M8 since Co deficiency seemed to determine the inhibition of the nitrile hydratase production either at the transcriptional or translational level [138]. Due to the high amino acid sequence similarity with Ni^2+^ HoxN transporter of *Alcaligenes eutrophus* [139], the Nh1F transporter was also proposed to promote the Ni^2+^ uptake in some actinobacterial members because of the metal-binding promiscuity [137], despite the preferential binding of Co^2+^. This aspect was further proven by the enhanced Ni-dependent urease activity showed by a recombinant strain of *E. coli* heterologously expressing the *Rhodococcus nhlF* gene and the successful cross-complementation of a *hoxN* deletion with the *nhlF* gene in *A. eutrophus* strain [140]. Moreover, Amoroso and coworkers (2000) highlighted that some of the 25 streptomycetes isolated from a uranium mining site displayed a great tolerance against Ni^2+^ ions (10 mM), among others (e.g., Co^2+^, Cd^2+^, Cu^2+^, Cr^3+^, and Zi^2+^) [141], likely due to the presence of a high-affinity Ni-transport system, which was identified in *Streptomyces* F4 and E8 strains using degenerated primer pairs designed on the basis of the Ni-transporter genes *hoxN* and *nixA* from *Ralstonia eutropha* [132] and *H. pylori* [142], respectively.

Zinc is another important transition metal that can act as a stabilizing element during protein folding and catalytic cofactor of several enzymes [143]. However, Zn poisoning can inhibit the electron transport chain and either block thiols or compete with other essential metal(loid)s for binding sites present in proteins, as reported in the case of cytochrome c oxidase [144,145]. Thus, bacteria must finely regulate and maintain Zn homeostasis [146]. To this aim, various microorganisms feature specific zinc regulators (Zur), which belong to the Fur family and are responsible for the regulation of genes devoted to Zn transport and homeostasis maintenance. In *S. coelicolor*, the Zn regulator Zur is located downstream of the *znuACB* operon transcribing for a putative Zn transporter. In particular, Zn could repress the transcript level of the *znuA* gene, while the deletion mutant in the *zur* gene did not show any repression. Moreover, the purified Zur protein could bind the *znuA* promoter as a homodimer in the presence of Zn, further highlighting the role of this repressor responsive to Zn [147]. Later, Choi and colleagues (2017) demonstrated that not only Zur negatively regulates genes encoding for proteins involved in Zn uptake, but it induces the expression of the *zitB* gene, which encodes for a Zn exporter in *S. coelicolor* [148]. At low Zn concentration (e.g., femtomolar), the regulator Zur binds, as a dimer, its box motif present in the upstream region of the *zitB* promoter, which results in a weak *zitB* expression. On the other hand, at high Zn concentrations (e.g., micromolar), oligomeric Zur binds its target region with a footprint that goes beyond the zinc box motif, resulting in an enhanced *zitB* induction [148], thus highlighting how a single metal regulator can maintain the intracellular free level of Zn within a narrow range by controlling the expression of genes coding for both uptake and export systems. Despite the fine regulation system for Zn homeostasis in bacteria, it is worth noting the good adaptability of some *Streptomycetes* strains to the high metal load, as reported in the case of *Streptomyces* K11, which could tolerate and bioaccumulate very high concentrations of Zn^2+^ ions [149].

Consequently, to metal(loid) bioaccumulation, microbial cells can show non-orthodox features such as electron-dense inclusions due to the localization of the sequestered metal(loid) within cellular components or their binding to metallothioneins [150,151]. For instance, electron-dense inclusions were visible through SEM microscopy in *Streptomyces* sp. MC1 cells as a possible result of the resistance mechanism of this strain to Cr^6+^ [152]. An additional effect of Cr was the generation of more rounded and shorter filamentous cells with respect to unexposed ones [153]. Further, some Actinobacteria members featured the ability to uptake radionuclides as Cs, whose presence on the Earth’s crust gained a global concern since the Chernobyl accident in 1986. In this regard, several rhodococci can cope with the toxicity derived from both Cs and rubidium (Rb), as reported for *R. erythropolis* CS98 and *Rhodococcus* sp. strain CS402 accumulating high levels of Cs, whose process was inhibited by both potassium (K) and Rb [154], probably because of the involvement of the poorly specific K-transport system [155,156].

### 2.3. Biotic Metal(loid) Reduction Reaction

The tolerance of bacteria to metal(loid) polluted environments is enhanced by reducing these chemical species into less toxic and bioavailable forms, guaranteeing the microbial survival under harsh conditions. Numerous studies reported on Actinobacteria’s capability of coping with metal(loid) cation and oxyanion toxicity through either intra- or extracellular bioconversion strategies (Table 3), being *Arthrobacter* and *Streptomyces* spp. considered as model microorganisms [14].

*Arthrobacter* and *Streptomyces* spp. can reduce, under different microbial growth conditions, Cr^6+^ (present in the form of chromate, CrO_4_^2−^) to the less harmful Cr^3+^ [14]. Similarly, Cr^6+^ reduction was also described for Actinobacteria belonging to *Microbacterium*, *Flexivirga*, *Intrasporangium*, *Corynebacterium*, *Micrococcus*, and *Amycolatopsis* spp. [152,160,180,199,201,210,214]. In *Arthrobacter* and *Streptomyces* spp., Cr^6+^ reduction can occur either intra- or extracellularly. In the latter, enzymes are produced and secreted by bacterial cells [210], while intracellular mechanisms generally implied the biosorption and uptake of metal cations, their reduction in the cytoplasm, and the accumulation of Cr^3+^ as intracellular deposits through metal binding with extracellular or intracellular proteins (Table 3) [153,210]. Moreover, Sugiyama and colleagues (2012) proposed the mechanism of Cr^6+^ bioreduction in *Flexivirga alba* ST13T, where the metal cations were first reduced either on the cell surface or intracellularly using glucose as an electron donor; the as-produced soluble Cr^3+^ ions were then extracellularly secreted and precipitate as Cr(OH)_3_ deposits, which bound the bacterial cell wall [180].

*Streptomyces* sp. 3 M and *A. crystallopoietes* ES 32 were the first members belonging to the corresponding species described to bioconvert Cr^6+^ through either a constitutive enzyme or an NADH-dependent chromate reductase [159,165]. A similar constitutive reduction mechanism was identified in *Streptomyces* sp. MC1, which featured a high Cr^6+^-reductase activity [174]. An intracellular enzymatic reduction appeared to be responsible for Cr^6+^ biotransformation in *S. siyoaensis* Lv81-138 [192], *A. rhombi*-RE [175], *A. ramosus* [178], and *A. aurescens* MM10 [179]. On the other hand, reducing substances of nonenzymatic nature mediated the bioconversion of Cr^6+^ into CrOOH in *S. thermocarboxydus* NH50. These reducing agents were released into the culture supernatants, and their production was induced upon the addition of the metal precursor [164,223].

In actinobacterial members, Cr^6+^ reduction depends on several factors, including the initial metal concentration, pH and temperature of the system, composition of the culture medium, physiological state and growth phase of the cells, as well as the addition of either cations or anions to the medium. Indeed, the higher the concentration of Cr^6+^ precursor, the lower is the rate of Cr reduction, likely due to increased toxicity of these cations [152,161,171,173,183,184,185,186,201]. The pH and the temperature of the microbial cultures differentially influenced Cr^6+^ bioconversion as a function of the actinobacterial species under analysis, revealing maximum reduction rates at optimal bacterial growth conditions [153,161,171,185,186,201]. It is noteworthy mentioning that pH variations from its optimum could change the ionization rate and conformation of the enzyme(s) devoted to Cr^6+^ bioprocessing, limiting their activity [171]. However, pH alterations from 4 to 9 in *Streptomyces* sp. MC1 cultures did not affect Cr^6+^ bioconversion, highlighting the stability of the enzymatic system involved [173]. On the other hand, the addition of glucose, yeast extract, or peptone to the culture media generally led to an increased Cr^6+^ reduction, as these carbon compounds acted as electron donors for both the uptake and transformation of metal cations and sources of reducing equivalents (i.e., NADH), supporting this bioprocess [152,153,161,164,171,174,180,183,184,185,199,201,210]. In this regard, the addition, at the initial time of incubation, of either organic compounds or metal ions to *S. griseus* resting cell suspension resulted in fast Cr^6+^ biotransformation [161] during *Streptomyces* sp. MC1 cultures showed a low rate of metal bioprocessing at prolonged Cr exposure times [153]. Providing either *Streptomyces* or *Arthrobacter* spp. culture media with diverse metal(loid) ions also influenced Cr^6+^ reduction. The addition of Cu^2+^ led to an increased rate of intra- and extracellular Cr^6+^ bioconversion, indicating that Cu could act as a “catalyst” for this bioprocess [164,183,184,185,186]. The presence of Co^2+^, Zn^2+^, and several anions in *Streptomyces* spp. growth media similarly enhanced Cr reduction [186], which was not influenced by Ni^2+^, Cd^2+^, and Pb^2+^ [164,186]. The only ion negatively affecting Cr reduction was Ca^2+^, likely due to a strong competition between these two metals for the binding site present in the enzymatic system(s) [171]. Conversely, divalent cations, such as Co^2+^, Hg^2+^, Ni^2+^, Cd^2+^, Zn^2+^, and Ba^2+^, greatly inhibited Cr^6+^ reduction in *Arthrobacter* spp., being Ca^2+^ and Cu^2+^ the only metal species that improved the rate of this bioprocess [175,183,184,185].

Besides Cr bioprocessing, several actinobacterial spp. strains showed a good ability in facing Hg^2+^, Cu^2+^, Ag^+^, Au^3+^, and SeO_3_^2−^ toxicity mostly through NAD(P)H-dependent reductases and dehydrogenases (Table 3), although a deep characterization of these bioprocesses is still lacking. Particularly, NADH-dependent reductases specific to this actinobacterial genus played a key role in Ag^+^ and Zn^2+^ bioconversion performed by *Rhodococcus* sp. NCIM2891 and *R. pyridinivorans* NT2, as proven by the high upregulation of these enzymes upon bacterial exposure to the metal precursor [187,193]. The extracellular α-amylase and the cell wall polysaccharide teichuronic acid (TUA) were instead responsible for Au^3+^ reduction in *M. luteus* NCIM 2379 [190]. Indeed, the partially purified enzyme was capable of bioconverting Au cations at 55 °C (optimum temperature for α-amylase), possibly through the reaction between Au^3+^ and thiol (RSH) groups of the amylase, while the reactive D-glucose and N-acetyl-D-mannosaminuronic acid moieties present within TUA were suggested to mediate this bioprocess in the heat-inactivated dried *M. luteus* biomass [190]. In *Actinobacter* sp., the observed rapid reduction of Au^3+^ was ascribed to the abundant secretion of the enzyme cytochrome oxidase, whose activity was enhanced in an anoxic environment at specific temperatures (i.e., 37 °C) [169,224]. The same environmental isolate also showed good proficiency in biotransforming Fe-containing compounds, as either mixture (ferricyanide (K_3_Fe(CN)_6_/ferrocyanide (K_4_Fe(CN)_6_), ferric chloride (FeCl_3_)/ferrous sulfate (FeSO_4_)) or single salts (FeCl_3_) [167,170]. The exposure of *Actinobacter* sp. to K_3_Fe(CN)_6_/ K_4_Fe(CN)_6_ mixture stimulated the induction of two proteins with hydrolyzing activity, likely responsible for Fe-bioconversion [167], while the presence of either FeCl_3_ or FeCl_3_/FeSO_4_ in the culture media triggered a separate cell response [170]. The bioreduction of Fe^3+^ within FeCl_3_ to the less toxic Fe^2+^ was mediated by an extracellular iron reductase with siderophore activity—i.e., ferrisiderophore reductase—whose concentration increased in response to the excess of Fe salt supplied to the growth medium [170]. The importance of these reductases in Fe bioprocessing was confirmed by the addition of Zn salts, as potent inhibitors of Fe reductases, to *Actinobacter* sp. cultures, which determined the lack of Fe-bioconversion [170]. Similarly, when the same environmental isolate was incubated in the presence of FeCl_3_/FeSO_4_ mixture, two additional proteins, identified as phosphoadenosyl sulfate and sulfite reductases, were detected, indicating their implication in handling the stress deriving from SO_4_^2−^ ions, which was confirmed by their competition with CrO_4_^2−^ [170].

In Actinobacteria, the reduction of metal(loid) oxyanions, such as SeO_3_^2−^, TeO_3_^2−^, and AsO_4_^3−^, is mostly mediated by RSH-containing molecules, which are abundantly present within bacterial cells [225], through the so-called Painter-type reaction [226]. According to the latest proposed mechanism for Se-oxyanion bioconversion [227], SeO_3_^2−^ react with RSH-containing compounds producing a relatively stable intermediate featuring Se^2+^ and superoxide ions (O_2_^−^), which undergo several biotransformation steps to prevent oxidative damage. The Se^2+^-intermediate is then bioconverted by Glutathione (GSH) or thioredoxin (Trx) reductases generating a highly unstable intermediate (i.e., RS-Se^−^) that spontaneously dismutates in Se^0^, favoring the regeneration of intracellular RSH-pool [227]. This mechanism was originally proposed for Gram-negative bacterial strains, as they feature high intracellular concentrations of GSHs, which are considered the first stress–response molecules for handling the presence of metal(loid) ions [227]. However, the abundance of MSHs in Actinobacteria and their greater redox stability as compared to GSHs [225] confers to these microorganisms the ability to handle and reduce high amounts of oxyanions [228]. In this regard, the Painter-type reaction appeared to be involved in either the intracellular or membrane-bound reduction of the oxyanions SeO_3_^2−^ and TeO_3_^2−^ to their elemental forms (Se^0^ and Te^0^) in both *Rhodococcus* and *Streptomyces* spp. [203,204,208,209], as well as the intracellular bioconversion of the oxyanion AsO_4_^3−^ to AsO_3_^3−^ by *C. glutamicum* [168,229] and *R. etherivorans* BCP1 [215] through the NADPH-dependent MSH_Mrx1 pathway [230,231]. In both these strains, a second yet important mechanism proposed for AsO_4_^3−^ bioconversion is based on the activity of thioredoxin- and NADPH-dependent ArsC proteins that mediate RSH/disulfide redox reactions [215,229].

### 2.4. Metal Efflux Systems

Besides the metal(loid) uptake, bacteria have evolved an extraordinary variety of resistance mechanisms that overall rely on multidrug efflux systems (MES) [232,233], which control the accumulation of molecules and their homeostasis between the intra- and extracellular environment. These efflux systems are essentially responsible for the protection of microorganisms against exceeding amounts of organic solvents, metal(loid)s, toxic lipids, quorum sensing molecules, and biocides, to name a few. The substrate specificity of most MES is very broad, thus representing the main factor contributing to the development of the microbial multidrug resistance (MDR) phenotype [234]. Based on the amino acid sequence identity and the energy source (either ATP hydrolysis or chemical gradient), MES can be distinguished into five main families: (i) ABC transporters, (ii) small multidrug resistance (SMR), (iii) resistance-nodulation cell division (RND), (iv) major facilitator superfamily (MFS), and (v) multidrug and toxic compound extrusion (MATE) [235,236,237,238]. The genome analysis approach carried out by Getsin and colleagues (2013) unveiled that *S. coelicolor* carries a total of 156 systems, which include channel pores, primary channels, secondary carries, translocators, transmembrane electron flow carriers, auxiliary proteins, and other poorly characterized putative transporters, devoted to both intra- and extracellular trafficking of inorganic compounds [239]. This study highlights the genome-based enzymatic systems inferred to have a role in metal import and efflux processes in this actinobacterial strain, therefore being involved in metal homeostasis and cell survival [239]. In this regard, when very high Ni^2+^ concentrations were present in the growth medium of *S. coelicolor* A3(2), the cells expressed the Ni-responsive regulator Nur, which is responsible for the downregulation of genes coding for enzymes involved in the Ni influx [135]. Yet, *S. coelicolor* A3(2) could grow only in media amended with low Ni concentrations [240], likely because of nonspecific Ni-transporters such as those involved in Mg^2+^ import [129], which mediate the uncontrolled entry of Ni^2+^ and may compromise the bacterial cell survival. Furthermore, Ni^2+^ accumulation supported the activity of Ni-dependent enzymes in *Streptomyces* F4 and E8 strains [141], while excesses of these cations became severely toxic [241]. In another example, *R. opacus* possesses a finely regulated uptake/efflux system dependent on Zn concentration, which is responsible for the Zn^2+^ uptake as efficient as the efflux one, thus maintaining physiological levels of these cations in the cell cytoplasm. To prove this point, Zn-loaded cells exposed to an additional amount of either Zn^2+^ or Cd^2+^ showed a decrease in the level of intracellular Zn^2+^, suggesting both an inducible (Zn^2+^) and a cross-inducible (Cd^2+^) metal efflux system [242].

Specific metal efflux systems are also involved in arsenic resistance mechanisms. Arsenic detoxification generally relies on the conversion of AsO_4_^3−^ to AsO_3_^3−^, which is further extruded from the cell using the arsenite efflux proteins ArsB or Acr3 [243]. The genomes of Actinobacteria almost exclusively possess genes encoding Acr3 permease as an arsenite extrusion system. In *C. glutamicum*, Acr3 mediated arsenite efflux depending on electrochemical energy [244]. On the other hand, in *R. etherivorans* BCP1, Acr3 seemed to be associated with the ATPase activity of the AsO_3_^3−^ transporting ArsA, resulting in the production of an efficient arsenite extrusion system represented by ATPase ArsA-Acr3 pump, which may contribute to the great resistance shown by this strain towards AsO_4_^3−^ [215].

## 3. Metal(loid) Nanomaterial Biosynthesis by Actinobacteria

**Figure 2:**
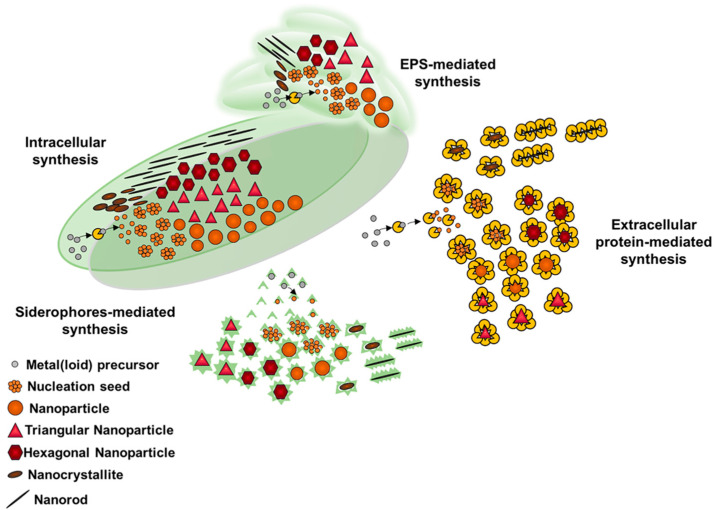
Schematic representation of the diverse biogenic nanomaterial morphologies obtained by actinobacterial biotransformation of metal salt precursors through intra- or extracellular (i.e., mediated by siderophores, extracellular proteins, and extracellular polymeric substances (EPS)) processes.

**Figure 3:**
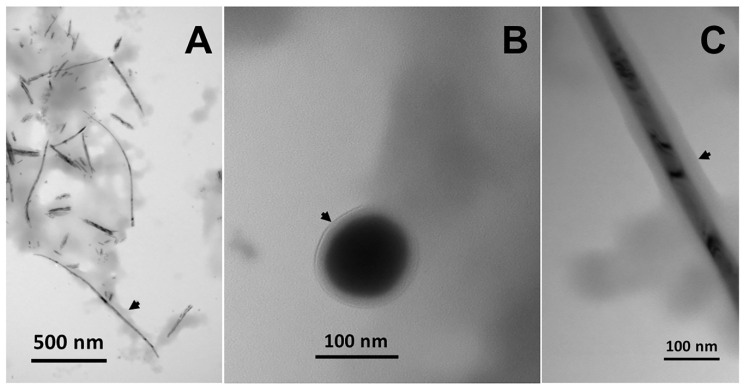
Biogenic nanomaterials in the form of tellurium nanorods (TeNRs) (**A**), selenium nanoparticles (SeNPs) (**B**), and selenium nanorods (SeNRs) (**C**) produced by the actinomycete *Rhodococcus etherivorans* BCP1 cells grown in liquid-rich medium amended with either potassium tellurite of sodium selenite as metal salt precursors. Arrowheads point toward the slight electron-dense organic coating featuring biogenically produced nanostructures.

Although diverse biochemical strategies could be exploited by bacteria to regulate metal(loid) concentration in the environmental niches they live in, these microorganisms elicit metabolic traits aimed at detoxifying their habitat, generally involving the reduction of metal(loid) ions to less toxic and bioavailable (e.g., elemental) forms. This process results in a high intra- or extracellular localized concentration of metal(loid) atoms, which, to counteract their thermodynamic instability, tend to aggregate each other, eventually assembling in defined and uniform metal(loid) nanostructures (MeNSs; Figure 2 and Figure 3) [162,204,208,209]. Thus, the proficiency of several bacterial genera, including Actinobacteria, to cope with metal(loid) ions makes them ideal candidates in the microbial nanotechnology field—i.e., the exploitation of microorganisms for the *green* and *eco-friendly* production of valuable Me nanomaterials (MeNMs). Indeed, a wide spectrum of Actinobacteria showed a strong attitude in biosynthesizing intra-, extracellular, or membrane-bound MeNMs with different sizes, morphologies, and properties (Table 4). However, parameters and biochemical processes influencing the production of MeNSs and their assembly, in combination with a lack of their extensive physical-chemical characterization and potential applications, are still the black holes of this emerging field, underlining some critical concerns about the possibility of scaling-up the biogenic route behind NMs production stream. In this regard, key aspects to consider in designing innovative MeNM production methods are the choice of reducing agents, metal(loid) precursor-to-reducing agent ratio, temperature, pH, and reaction time. Considering the biogenic synthesis, controlling the reducing agent(s) is a very demanding task, especially when whole cells or EPS are used, as several and complex biomolecules could participate in these bioprocesses [22]. Moreover, whole-cell mediated synthesis also forces to keep constant the temperature and pH of the system, parameters that are optimized for the bacterial strain investigated. Particularly, a temperature between 30 and 37 °C and pH values close to neutrality are the optimum conditions to favor the growth of Actinobacteria [169]. Alternatively, when the metal(loid) reduction occurs through cell-free extracts or EPS, a higher flexibility for these two parameters can lead to an improved optimization of the NM production. For instance, a temperature of 70–100 °C mediated the fast reduction of Au^3+^ or Ag^+^ generating MeNSs using cell-free extracts derived from *Nocardia farcinica* [182] and *Gordonia amarae* [202], as well as the EPS recovered from *Arthrobacter* sp. B4 [245].

Changes in the pH can differentially influence MeNM production, depending on the functional groups present within the bacterial extracts. An acidic pH value (ca. 4) allowed to obtain monodispersed and uniform AuNPs by *N. farcinica* extract [182], while the EPS extracted from *Arthrobacter* sp. B4 [245] and *G. amarae* [202] required a pH either close to neutrality (7–8) or alkaline conditions (10–12) for the reduction of Ag^+^ or Au^3+^, respectively, leading to the corresponding MeNM production. Extracting specific biomolecules—i.e., glycolipids [253], α-amylases, and TUA [190]—guarantees better control over both the mechanism responsible for the metal(loid) reduction and the concentration of the reducing agent available. Nevertheless, strict control of temperature and pH is still needed to assure the biomolecules’ functional activity [190]. Another parameter influencing the production of biogenic MeNMs is the metal(loid) precursor concentration, which exerts a positive effect until a certain threshold value—i.e., the maximum amount of metal(loid) ions that can be reduced by the actinobacterial strain—is reached [202]. The effect of the precursor-to-reducing agent ratio on the biogenic MeNS synthesis was explored in the case of AgNPs produced by the EPS extracted from *Arthrobacter* sp. B4 cultures, for which the addition of a large EPS amount determined a high rate of NP synthesis, likely due to an enhanced number of reactive groups interacting with Ag^+^ [245]. A similar relationship was observed for resting cells belonging to *Rhodococcus* and *Gordonia* genera incubated for different time intervals in the presence of metal(loid) ions, as the adaptation of these microorganisms to these chemical species seemed to mediate a faster ion bioconversion, leading to MeNM generation [202,209]. However, these reduction reactions generally reach a plateau that is related to the redox stability of microbial reduction systems, reflecting the bacterial tolerance towards the metal(loid) challenge [202]. Further, increasing the precursor concentration and/or the time of incubation can induce (i) a morphological transition of MeNMs, determining the generation of cubic or hexagonal NPs and one-dimensional nanorods (NRs), as well as (ii) the growth and elongation of NPs and NRs, consequently to the increased density of elemental atoms that can be reached within the infinitesimal spatial volume represented by the bacterial cytoplasm, the latter being true for those strains capable of generating intracellular MeNMs [22,167,209]. This aspect was studied in detail in the case of TeNRs produced by *R. etherivorans* BCP1 cells under different conditions [204,209]. Stirring and oxygen flow rates were also parameters to account for the synthesis of Fe_3_O_2_ NPs by *R. erythropolis* ATCC 4277 grown in a tank reactor [218,219]. Indeed, a high, stirring rate resulted in large intracellular NPs due to the prevention in releasing enzymes and proteins generally responsible for the extracellular NP synthesis, while the lack of dissolved oxygen may have forced the production of reductases, enhancing NP generation [219]. Similarly, when the reduction of chloroauric acid (HAuCl_4_) by *Actinobacter* sp. was carried out under anoxic conditions, the rate of the reaction itself was enhanced, and the as-produced AuNPs were more uniform in shape and monodisperse in size [169].

The inherent complexity and diversity of a biological system, such as that of a bacterial cell, unveils the importance of choosing the microbial cell factory to use, as well as its cell physiology, to develop biogenic MeNM synthetic processes. Different actinobacterial strains have diverse capabilities of processing metal(loid) ions and producing MeNMs, whose cell localization, size, and shape can greatly change in a given microorganism depending on its growth conditions (Table 4). Such an effect can be achieved by playing with the bacterial cell physiology; for instance, *R. etherivorans* BCP1 cells exposed twice (i.e., *conditioned*) to the same metal(loid) precursor (SeO_3_^2−^ or TeO_3_^2−^) showed a higher rate of oxyanion bioconversion as compared to those exposed once, causing an enlargement or an elongation of SeNPs or TeNRs [204,208], respectively. Nevertheless, the highest reduction rate was detected upon the use of BCP1 resting cells to synthesize long and uniform TeNRs, likely due to the high initial redox potential of these bacterial cells [209]. On the other hand, bacterial strains growing in a minimal growth medium supplied with a single source of carbon could result in a reduced metal(loid) ion removal efficiency, probably due to a lower amount of reducing equivalents available that can support the biotic metal(loid) transformation as compared to bacterial cells growing in complex and rich media. However, the bacterial growth on a minimal medium can give rise to more homogeneous MeNMs, representing an advantage from a biotechnological point of view [275].

Although actinobacterial strains and corresponding cell-free extracts efficiently produce MeNMs, the mechanism(s) governing their assembly in the intra- or extracellular environment is still poorly understood. Some insights were put forward by studying the capability of *R. etherivorans* BCP1 cells of biosynthesizing Se or TeNMs [204,208,209]. In this regard, the formation of these NSs occurred following the so-called LaMer mechanism, by which, once a critical concentration of Se or Te atoms is reached, these atoms collapse, generating nucleation seeds. The latter then undergo aggregation when a critical concentration value is reached, which determines the formation of amorphous Se or Te NPs. Since these NPs feature high surface energy and are confined in the intracellular environment, they tend to dissolve and deposit along one axis, forming more thermodynamically stable Se or TeNRs. This process is favored by the presence of amphiphilic biomolecules surrounding the forming NRs, which could act as a template for the mono-dimensional growth of these NMs [204,208,209].

A common feature and a first indication of the microbial ability to produce MeNMs is the change in color [depending on the considered metal(loid)] generally observed within bacterial cultures or cell-free extracts, which is due to the arising of surface plasmon resonance (SPR) or SPR-like phenomena. Following the bioconversion of metal(loid)s, the reduced chemical species, as well as the forming NMs, generally develop unique optical properties as a function of the metal(loid) element, size, and morphology of the as-produced NSs [169,202,269,276]. The color variation of the suspensions can also be independent of SPR, as in the case of Fe_2_O_3_ NPs, which, depending on their size and crystalline configuration, can acquire red, brown, or black shades [277].

Regardless of the color change, the physical-chemical characterization of MeNMs produced by Actinobacteria revealed their versatility in synthesizing a broad spectrum of NSs with diverse localization, morphologies, size, crystalline configuration, and, therefore, properties, whose overview is presented in Table 4. Among these features, the localization of biogenic MeNSs plays a fundamental role in terms of the easiness of their recovery, being the extracellular production preferred, as it makes it unnecessary to perform expensive and time-consuming procedures, such as the isolation of MeNSs from the cell cytosol [21,22]. However, stable and crystalline MeNRs are generally obtained through intracellular processes [208,209], likely due to the high local concentrations of elemental atoms available for their deposition along one axis. Thus, depending on the desired final product and the actual feasibility of the bioprocesses, different actinobacterial strains can be exploited for NM production, which can be optimized considering the above-mentioned parameters.

Another important physical-chemical feature of biogenic NMs is their high thermodynamic stability under diverse conditions, which results in the absence of aggregation phenomena, avoiding the need for postproduction treatments to ensure the applicability of these NSs [22]. This key aspect is to be traced back to the presence of organic material within the biogenic NM extracts that contain biomolecules either derived from or secreted by actinobacterial cells (Table 4). These biomolecules, mostly represented by proteins and enzymes, but also by polypeptides, glycolipids, secondary metabolites, and amphiphilic compounds (Table 4), can reduce the metal(loid) precursor (Table 3) as well as thermodynamically stabilize the forming NMs, through reactive functional groups and steric hindrance phenomena [22]. These chemical features ensure the development of both electrostatic (between reactive groups) and steric (due to the bulky structure) interactions between the biomolecules themselves and MeNSs, decreasing their high surface energy and conferring them good thermodynamic stability [22]. The nature and strength of these interactions seemed to control size, shape, crystalline configuration, and aggregation of Au, Ag, and ZnO NPs produced by *Rhodococcus* spp., *N. farcinica*, *Rhodococcus* NCIM 2891, and *R. pyridinivorans* NT2 [163,182,193,257]. Indeed, biomolecules capping AgNPs recovered from *Rhodococcus* NCIM 2891 conferred to these NMs strong stability in neutral, alkaline, and slightly acidic conditions. At alkaline pH values, COO^−^ groups were fully charged and could mediate strong electrostatic repulsive interactions, which, however, disappeared as the pH became closer to the pK_a_ (ca. 5) of this reactive functional group [257]. Similarly, these biogenic AgNPs were thermodynamically stable up to 60 °C, the temperature at which the surrounding biomolecules may denature, hence determining their inactivation [257]. Since several washing steps were not sufficient to completely remove the organic material from the biogenic MeNM extracts recovered from *Rhodococcus* NCIM 2891, *R. pyridinivorans* NT2, and *R. etherivorans* BCP1 [193,204,208,209], it was suggested that thermodynamic equilibrium is reached between the biomolecules and NSs, thus allowing a strong stabilization of the latter. To further support this hypothesis, Kundu et al. (2014) compared the thermodynamic stability of ZnO NPs produced by *R. pyridinivorans* NT2 with those commercially available. This indicated that the incubation of these NPs with bacterial extracellular proteins favored their stabilization overtime. Indeed, ZnO NPs synthesized by NT2 cells remained steadily dispersed in water for more than 2 months, while the chemogenic ones revealed lower stability [193].

### Properties and Applications of Metal(loid) NMs Produced by Actinobacteria

The quality of biogenic MeNMs allows their use in the (bio)technological field, as they show promising antibacterial, antifungal, antioxidant, antiviral, acaricidal, larvicidal, anti-biofouling, anticancer, electrochemical, fluorescent, conductive, superparamagnetic, (photo)catalytic, and sensory properties, to name a few (Table 4).

Currently, resistance to antibiotics by microorganisms (AMR) is a major problem not easy to solve; hence, the potential of biogenic MeNMs, such as AgNPs, to act as bacteriostatic and/or bactericidal compounds has gained an impressive scientific and technological interest [278]. Overall, AgNPs produced by Actinobacteria displays a higher antibacterial activity against Gram-negative as compared to Gram-positive bacteria, likely due to their thicker peptidoglycan layer [189]. Furthermore, biogenic AgNPs can prevent pathogen biofilm formation [200,270], becoming largely promising for the treatment of hospital-oriented infections. Although several studies reported on the antimicrobial potential of biogenic AgNPs, alongside other MeNMs, three possible mechanisms have been formulated to date to explain their antimicrobial efficiency: (i) NP attachment to the cell membrane negatively affecting its bioenergetic functions (i.e., permeability and respiration), (ii) NP penetration within the cells by interacting with S- and P-containing compounds (i.e., DNA) causing lethal damage and the depletion of ATP intracellular reservoir, or (iii) the release of Ag^+^, causing DNA to lose its replication ability and cellular proteins to become inactivated [189,279]. AgNPs, as well as SeNPs, have also been proven to be efficient, acting in synergy with antibiotics, enhancing the antimicrobial activity of the latter [189,200,222,265], as multiple resistance mechanisms are needed for the pathogens to simultaneously counteract these bioactive substances. Moreover, antibiotics contain many functional groups that can react with AgNPs, favoring their efficacy [189]. Similarly, Cu-, Zn-, and Se-based NPs produced by Actinobacteria featured high antimicrobial ability in limiting or preventing the proliferation of pathogens as either planktonic cells or biofilms [193,195,213]. In this regard, Cu-based NPs caused the production of hydroxyl radicals, which in turn determine the destruction of plasmid and genomic DNA and the inactivation of essential enzymes through their binding with Cu^2+^ [213,216]. Additionally, Cu-based NPs showed good larvicidal properties, as their size guarantees them an easy penetration through either the oral canal or the breaching of the larval cuticle membrane, leading to their diffusion within the body. Here, Cu-based NPs can bind, as for bacterial cells, to S- and P-containing biomolecules, causing inhibition of both DNA and protein synthesis [213].

Besides antimicrobials, common features of MeNMs obtained from Actinobacteria are their good anticancer and antioxidant properties (Table 4), yet their mode of action is still poorly understood, as they showed both antioxidant and pro-oxidant behaviors, as a function of the experimental conditions tested [198]. Indeed, although some of these NMs were described to induce apoptosis and cell cycle arrest [196], the most accredited mechanism explaining the anticancer activity of biogenic MeNSs is their ability to promote the generation of ROS in cancer cells, eventually causing cell death. For this situation to happen, a high level of free Cu^2+^ is needed in the cellular environment, as they can bind with NMs (e.g., SeNPs) along with DNA, forming a ternary complex, which can undergo a redox reaction that determines the generation of Cu^+^ ions, whose subsequent oxidation produces ROS [198]. Since an elevated level of Cu^2+^ is quite common within cancer tissues and cells, the mobilization of these ions can cause the pro-oxidation activity of MeNMs [198]. On the other hand, in normal conditions, these NSs exhibit strong antioxidant activity by functioning as free radical scavengers for ROS in living organisms [196,216,253]. The morphology and the size of biogenic MeNMs are the two parameters highly influencing this antioxidant behavior, being monodispersed NPs more suitable for this task [216,253]. All these properties are even emphasized by the low cytotoxicity of biogenic MeNMs towards human cell lines [193,207,216,270], encouraging the potential applications of these nanoformulations in the biomedical field.

From a technological point of view, Au, Ag, ZnO, and Te NMs produced by the Actinobacteria featured good electrochemical [176], (photo)catalytic [193,254,257], sensing [202], superparamagnetic [167,170,171], and conductive [176,208] properties (Table 4). Particularly, AuNPs synthesized by *G. amarae* extract combined with the amino acid L-cysteine revealed a high proficiency as a sensor for Cu^2+^ at concentrations as low as 4.0 nM [202]. The addition of L-cysteine to AuNP solutions determines the aggregation of the NPs through -SH groups on the amino acid; however, when Cu^2+^ are present, L-cysteine undergoes an oxidation reaction becoming cystine, in which any free -SH moiety is not available to interact with AuNPs [202]. Since the aggregation of AuNPs is linked to their SPR phenomena, a change in the absorption profile of NPs themselves occurs, making the interaction between L-cysteine and Cu^2+^ easily detectable through UV-visible spectroscopy [202]. Moreover, upon addition of other metal ions, AuNP agglomeration was still detected, indicating the specificity of AuNPs recovered from *G. amarae* cell-free extract for the detection of Cu ions in suspension without the need of additional tags or functionalization with specific ligands [202].

## 4. Conclusions

Several members of the Actinobacteria phylum are capable of withstanding and converting toxic metals into harmless forms ascribed to metabolism-dependent and -independent cellular activities. Thus, a better comprehension of these biotic processes constitutes an important basis for developing successful bioremediation approaches to reclaim metal(loid)-polluted environments by using either actinobacterial pure cultures or ad hoc consortia, which meet the requested criteria of *green* chemistry and circular economy. Moreover, a relevant added value of these bioprocesses is represented by the proficiency of Actinobacteria to bioconvert toxic metal(loid) ions, consequently producing nanomaterials of various sizes, shapes, and composition that feature a broad spectrum of applicative physical-chemical (e.g., optical, photoluminescent, photocatalytical, electrical) and biological (anticancer and antimicrobial) properties. Nevertheless, the knowledge regarding (i) biological mechanisms behind metal(loid) nanostructure production, (ii) the influence of cell physiology and metabolism on the final nanoscaled products, (iii) the optimization of nanomaterial recovery from actinobacterial cultures or extracts, (iv) the identity of macromolecules determining the high thermodynamic stabilization of these nanostructures, and (v) their structure-to-properties relationships still needs to be improved to guarantee the translation of biogenic nanostructures from benchtop research to their practical application.

## Figures and Tables

**Table 1:** Siderophores produced by Actinobacteria as metal(loid) chelating agents.

Actinobacteria Strains	Siderophores	Metal Tolerance	References
*Microbacterium flavescens* JG-9	Desferrioxamine-B	U and Pu	[59]
*Arthrobacter* spp., *Microbacterium* spp., *Curtobacterium* spp., *Leifsonia* spp.	N.D. ^1^	Co, Cr, Cu, Ni, Zn	[60]
*Streptomyces* sp. AR16 and AR36	N.D. ^1^	Zn, Cd, Pb	[61]
*Microbacterium* G16	N.D. ^1^	Cu, Cd, Ni, Pb, Zn	[62]
*Streptomyces acidiscabies* E13	Coelichelin and desferrioxamines	Ni	[63]
*Streptomyces tendae* F4	Coelichelin and desferrioxamines	Ni, Cd	[64,65]
*Methylobacterium* and *Frigobacterium* spp.	N.D. ^1^	Zn, Cd	[66]
*Arthrobacter* spp. and *M. kitamiense* YM31	N.D. ^1^	Cu	[67]
*Microbacterium* sp. NCr-8, *Arthrobacter* sp. NCr-1, *Kokuria* sp. NCr-3	N.D. ^1^	Ni	[68]
*Streptomyces mirabilis* P16B-1	Linear and cyclic ferrioxamines	Zn, Al, Ni, Cu	[69]
*Streptomyces* spp.	Hydroxamates	Pb	[70]
*Streptomyces* spp.	Ferrioxamines, phenolates, and catecholates	Cd	[71]
*Arthrobacter oxydans* ATW2 and ATW3, *Kocuria rosea* ATW4, *Rhodococcus erythropolis* ATW1 and S43	N.D. ^1^	As	[72]
*Rothia mucilaginosa* ATCC 25,296	Enterobactin	Zn, Mg	[73]

^1^ N.D. indicates not determined, which is referred to the nature of siderophores produced by Actinobacteria.

**Table 2:** Extracellular polymeric substances (EPS) produced by Actinobacteria as biosorbents for metal(loid)s.

Actinobacteria Strains	EPS Features	Metal Tolerance	References
*Arthrobacter* D9	N.D. ^1^	Cd	[91]
*Amycolatopsis* sp. AB0	Polysaccharides containing glucose	Cu	[92]
*Kocuria rizophila* DE2008	Polysaccharides and uronic acid	Cu, Pb	[93,94]
*Arthrobacter viscosus*	N.D. ^1^	Cr	[95]
*Nocardia amarae*	N.D. ^1^	Na, Ca, Al, Fe	[96]
*Arthrobacter* ps-5	Polysaccharides containing glucose and galactose	Pb, Cu, Cr	[81]
*Rhododoccus opacus* and *Rhodococcus rhodochrous*	N.D. ^1^	Ni, Cd, Pb, Co, Cr	[86]
*Kocuria rizophila* BPB1	N.D. ^1^	As	[97]
*Frankia* sp. strain EAN1pec	Polysaccharides and proteins	Pb	[98]
*Kokuria* sp. BRI 36	Polysaccharides and uronic acid	Pb, Cd, Cr	[99]
*Nocardiopsis* sp. 13H	Polysaccharides, proteins, and nucleic acids	Sr and Cs	[87,100]
*Streptomyces* sp. CuOff24	Arabinose, galactose, mannose, glucose, and uronic acid	Sr	[88]
*Glutamicibacter halophytocola* KLBMP 5180	Rhamnose, glucuronic acid, glucose, galactose, xylose, and arabinose	Mn, Cu, Fe, Ca, Mg	[83]

^1^ N.D. indicates not determined, which is referred to the nature of EPS produced by Actinobacteria.

**Table 3:** Overview of metal(loid) reduction processes in Actinobacteria.

Actinobacteria Strains	Metal(loid) Reduction	Mediating Biomolecules	References
*Micrococcus lactilyticus*	Au^3+^, Ag^+^, SeO_3_^2−^, TeO_3_^2−^, TeO_4_^2−^, AsO_4_^3−^, bismuthate (BiO_3_^−^), vanadate (VO_3_^4−^), molybdate (MoO_4_^2−^), ruthenium (Ru^3+^)	N.D. ^1^	[157]
*Streptomyces lividans* 1326	Hg^2+^	Hg-constitutive reductase	[158]
*Streptomyces* sp. 3 M	CrO_4_^2−^	Constitutive reductase	[159]
*Microbacterium* sp. MP30	CrO_4_^2−^	N.D. ^1^	[160]
*Streptomyces griseus*	CrO_4_^2−^	N.D. ^1^	[161]
*Thermomonospora* sp.	Au^3+^	Four proteins and enzymes (80–100 kDa)	[162]
*Rhodococcus* sp.	Au^3+^	Intracellular proteins	[163]
*Streptomyces thermocarboxydus* NH50	CrO_4_^2−^	Extracellular “nonenzymatic” substance	[164]
*Arthrobacter crystallopoietes* ES 32	CrO_4_^2−^	NADH-dependent chromate reductase	[165]
*Corynebacterium* strain SH09	Ag^+^	Aldehyde and ketone groups of biomolecules	[166]
*Actinobacter* sp.	K_3_Fe(CN)_6_/ K_4_Fe(CN)_6_ mixture	Extracellular hydrolases	[167]
*Corynebacterium glutamicum*	AsO_4_^3−^	NAD(P)H-dependent intracellular ArsC proteins	[168]
*Streptomyces* spp., *Amycolatopsis* sp.	CrO_4_^2−^	N.D. ^1^	[152]
*Actinobacter* sp.	Au^3+^	Cytochrome oxidase	[169]
*Actinobacter* sp.	FeCl_3_ or FeCl_3_/FeSO_4_ mixture	Ferrisiderophore reductase, phosphoadenosyl sulfate, and sulfite reductases	[170]
*Streptomyces* sp. MS-2	CrO_4_^2−^	N.D. ^1^	[171]
*Brevibacterium casei* AP6	Co^2+^	Intra- and extracellular proteins	[172]
*Streptomyces* sp. MC1	CrO_4_^2−^	NAD(P)H-dependent constitutive chromate reductase	[153,173,174]
*Arthroobacter rhombi*-RE	CrO_4_^2−^	Intracellular enzymes	[175]
*Streptomyces hygroscopicus*	Ag^+^	Secondary metabolites	[176]
*Corynebacterium glutamicum*	Ag^+^	Proteins	[177]
*Arthrobacter ramosus*	Hg^2+^, CrO_4_^2−^	MerA enzyme, intracellular enzymes	[178]
*Arthrobacter aurescens* MM10	CrO_4_^2−^	Intracellular chromate reductase	[179]
*Flexivirga alba* ST13	CrO_4_^2−^	Intracellular proteins	[180]
*Streptomyces* sp.	Ag^+^	Intracellular enzymes	[181]
*Nocardia farcinica*	Au^3+^	extracellular nitrate reductase	[182]
*Arthrobacter* sp. SUK 1201	CrO_4_^2−^	N.D. ^1^	[183,184]
*Arthrobacter* sp. SUK 1205	CrO_4_^2−^	N.D. ^1^	[185]
*Streptomyces* sp. RSF17, CRF14, *Streptomyces matansis* BG5, *Streptomyces vinaceus* CRF2, *Streptomyces pulcher* CRF17, *Streptomyces griseoincarnatus* SCF18	CrO_4_^2−^	N.D. ^1^	[186]
*Rhodococcus* NCIM 2891	Ag^+^	Intracellular NADH-dependent nitrate reductase; peptides, proteins, and carbohydrates	[187,188]
*Streptomyces* sp. JAR1	Ag^+^	Intracellular NADH-dependent nitrate reductase	[189]
*Micrococcus luteus* NCIM 2379	Au^3+^	Extracellular α-amylase and TUA	[190]
*Streptomyces* sp.VITDDK3	Au^3+^	(2S,5R,6R)-2-hydroxy-3,5,6-trimethyloctan-4-one	[191]
*Streptomyces sioyaensis* Lv81-138	CrO_4_^2−^	Intracellular reductases	[192]
*Rhodococcus pyridinivorans* NT2	Zn^2+^	NAD(P)H-dependent reductase and secreted reductase	[193]
*Streptomyces* spp. and *Amycolatopsis tucumanensis*	CrO_4_^2−^ and lindane	N.D. ^1^	[194]
*Kocuria flava* M-7	Cu^2+^	Intracellular proteins	[195]
*Streptomyces minutiscleroticus* M10A6	SeO_3_^2−^	Intracellular proteins	[196]
*Acidithrix ferrooxidans* PY-F3	Fe^3+^	N.D. ^1^	[197]
*Streptomyces bikiniensis* strain Ess_amA-I	SeO_3_^2−^	Intracellular proteins and enzymes	[198]
*Intraspongium chromatireducens* Q5-1	CrO_4_^2−^	Extracellular constitutive enzyme	[199]
*Streptomyces rochei* MHM13	Ag^+^	Intracellular proteins	[200]
*Corynebacterium paurometabolum* SKPD 1204	CrO_4_^2−^	N.D. ^1^	[201]
*Gordonia amarae*	Au^3+^	Glycolipids	[202]
*Streptomyces* sp. ES2-5	SeO_3_^2−^	Mycothiols and thiol-containing molecules	[203]
*Rhodococcus etherivorans* BCP1	TeO_3_^2−^	Mycothiols and thiol-containing molecules	[204]
*Streptomyces* sp. NH21	Au^3+^, Ag^+^	Intra- and extracellular proteins	[205]
*Streptomyces kasugaensis* M338-M1T, *Streptomyces celluloflavus* NRRL B-2493^T^	Ag^+^	Proteins	[206]
*Streptomyces parvulus* DPUA 1549, *Streptomyces owasiensis* DPUA 1748	Ag^+^	Proteins	[207]
*Rhodococcus etherivorans* BCP1	SeO_3_^2−^	Mycothiols and thiol-containing molecules	[208]
*Rhodococcus etherivorans* BCP1	TeO_3_^2−^	Mycothiols and thiol-containing molecules	[209]
*Streptomyces* spp. M7, A5, and MC1, *Amycolatopsis tucumanensis*	CrO_4_^2−^	N.D. ^1^	[210]
*Streptomyces* sp. Al-Dhabi 89	Ag^+^	Extracellular biomolecules	[211]
*Streptomyces xinghaiensis* OF1	Ag^+^	Organic compounds featuring amino bonds	[212]
*Streptomyces capillispiralis* Ca-1	Cu^2+^	Intracellular proteins	[213]
*Micrococcus luteus* HM 2 *and* HM 16	CrO_4_^2−^	Extracellular proteins	[214]
*Rhodococcus etherivorans* BCP1	AsO_4_^3−^	Mycothiols and NAD(P)H-dependent intracellular ArsC proteins	[215]
*Streptomyces zaomyceticus* Oc-5, *Streptomyces pseudogriseolus* Acv-11	Cu^2+^	Biomolecules containing peptide bonds, -NH, -OH, -CN, and C=C reactive groups	[216]
*Frankia inefficax* strain EuI1c	SeO_3_^2−^	NADH-dependent reductase and dehydrogenase	[217]
*Rhodococcus erythropolis* ATCC 4277	Fe^3+^	N.D. ^1^	[218,219]
*Streptomyces spongiicola* AS-3 (cell-free extracts)	Ag^+^	Proteins, alcohols, and terpenoids	[220]
*Nocardiopsis. dassonvillei*-DS013	Ag^+^	Biomolecules containing -OH, -C+O, -CCl, -CBr, and -C≡C reactive groups	[221]
*Streptomyces* sp. M10A65	SeO_3_^2−^	Intracellular NAD(P)H-dependent reductase	[222]

^1^ N.D. indicates not determined, which is referred to the nature of the biomolecules mediating metal(loid) reduction in Actinobacteria.

**Table 4:** Overview of metal(loid) NMs produced by Actinobacteria, their thermodynamic stabilization, physical-chemical and applicative properties.

Actinobacteria	Growth Conditions	Shape	Size (nm)	Crystal Structure	Stabilization	Properties	References
**AuNMs**							
*Thermomonospora* sp.	growing cells	AuNPs ^1^	ca. 10	fcc ^2^	extracellular secreted proteins	N.D. ^3^	[162]
*Rhodococcus* sp.	resting cells	AuNPs	ca. 9	fcc	N.D.	N.D.	[163]
*Actinobacter* sp.	BSA-exposed biomass	triangular hexagonal AuNPs	50–500	fcc	extracellular proteases	N.D.	[169]
	BSA-exposed biomass (anoxic)	triangular AuNPs	30–50	fcc			
	BSA-exposed biomass 25 C	AuNPs	N.D.	fcc			
*Streptomyces viridogens* HM10	resting cells	AuNPs	18–20	fcc	N.D.	antibacterial	[246]
*Arthrobacter* sp. 61B; *Arthrobacter globiformis* 151B	resting cells	AuNPs	8–40	fcc	N.D.	N.D.	[247]
*Streptomyces griseus*	resting cells	AuNPs	50	fcc	N.D.	N.D.	[248]
*Nocardia farcinica*	cell-free extract	AuNPs	15–20	fcc	N.D.	N.D.	[182]
*Streptomyces hygroscopicus*	resting cells (neutral pH)	AuNPs	2–10	fcc	intracellular bioactive compounds	antibacterial, electrochemical	[176]
*Streptomyces hygroscopicus*	resting cells (acidic pH)	hexagonal, pentagonal AuNPs	30–1500	fcc	intracellular bioactive compounds	antibacterial, electrochemical	[176]
*Streptomyces* sp. ERI-3	cell-free extract	AuNPs	ca. 9	fcc	N.D.	N.D.	[249]
*Micrococcus luteus* NCIM 2379	extracted α-amylase	AuNPs	ca. 6	N.D.	extracted α-amylase	N.D.	[192]
	extracted TUA	hexagonal AuNPs	ca. 50		extracted TUA		
*Streptomyces* sp. VITDDK3	cell-free extract	hexagonal, cubical AuNPs	ca. 90	fcc	(2S,5R,6R)-2-hydroxy-3,5,6-trimethyloctan-4-one	antifungal	[191]
*Sacchomonospora* sp.	cell-free extract	triangular AuNPs	40–80	fcc	polypeptides	N.D.	[250]
*Streptomyces hygroscopicus*	resting cells	AuNPs	10–20	N.D.	N.D.	N.D.	[251]
*Arthrobacter nitroguajacolicus*	resting cells	AuNPs	ca.40	fcc	N.D.	N.D.	[252]
*Gordonia amarae*	cell-free extract	AuNPs	15–40	fcc	N.D.	sensor for Cu detection	[202]
*Gordonia amicalis* HS-11	cell-free extract	AuNPs	5–25	fcc	glycolipids	antioxidant	[253]
*Streptomyces grisoruber*	cell-free extract	triangular, hexagonal AuNPs	5–50	fcc	extracellular biomolecules	catalytic	[254]
*Streptomyces* sp. NH21	cell-free extract	AuNPs	10	fcc	proteins	antibacterial	[205]
*Nocardiopsis dassonvillei* DS013	growing cells	AuNPs	30–80	fcc	proteins	antibacterial	[221]
**AgNMs**							
*Corynebacterium* strain SH09	resting cells	AgNPs	10–15	fcc	proteins	N.D.	[166]
*Streptomyces hygroscopicus*	cell-free extract	AgNPs	20–30	fcc	extracellular biomolecules	antibacterial	[176]
*Corynebacterium glutamicum*	resting cells	irregular AgNPs	5–50	fcc	proteins	N.D.	[177]
*Streptomyces glaucus* 71MD	resting cells	AgNPs	4–25	fcc	N.D.	N.D.	[255]
*Streptomyces* sp.	cell-free extract	AgNPs	15–25	fcc	extracellular proteins	N.D.	[181]
*Streptomyces rochei*	cell-free extract	N.D.	N.D.	N.D.	N.D.	antibacterial	[256]
*Rhodococcus* NCIM 2891	growing cells cell-free extract	AgNPs	ca.10	fcc	N.D.	N.D.	[187]
			5–50		proteins	antibacterial	[257]
			ca. 15		mesophilic proteins	fluorescent, antimicrobial, catalytic	[188]
*Nocardiopsis* sp. MBRC-1	cell-free extract	AgNPs	30–80	fcc	proteins	antimicrobial, anticancer	[258]
*Actinobacteria* sp. PSBVIT-13	cell-free extract	AgNPs	ca. 45	fcc	proteins	antibacterial	[259]
*Streptomyces* sp. JAR1	cell-free extract	N.D.	ca. 68	fcc	extracellular proteins	antibacterial, antifungal	[189]
*Micrococcus luteus*	growing cells	AgNPs	<100	fcc	N.D.	N.D.	[260]
*Streptomyces* sp. P-311	crude enzyme extract	AgNPs	100–200	fcc	N.D.	antibacterial	[261]
*Streptomyces* sp., *Streptoverticillium* sp.	growing cells	AgNPs	ca. 8 < 70	N.D.	N.D.	N.D.	[262]
*Streptomyces* sp. LK3	cell-free extract	AgNPs	ca. 5	fcc	N.D.	acaricidal	[263]
*Streptomyces* sp. SS2	cell-free extract and resting cells	AgNPs	ca. 67	N.D.	secondary metabolites	antibacterial	[264]
*Pilimelia columellifera* subsp. *pallida* SF23 and C9	cell-free extract	AgNPs	4–36 8–60	N.D.	extracellular proteins	antifungal	[265]
*Corynebacterium glutamicum*	resting cells	AgNPs	15	N.D.	N.D.	antibacterial	[266]
*Streptomyces rochei* MHM13	cell-free extract	AgNPs	22–85	N.D.	proteins	antibacterial, antibiofouling, anticancer	[200]
*Gordonia amicalis* HS-11	cell-free extract	AgNPs	5–25	fcc	glycolipids	antioxidant	[253]
	purified glycolipid		ca. 20				
*Streptomyces* sp. M-13 and M-24	cell-free extract	AgNPs	10–20	N.D.	N.D.	antibacterial	[267]
*Streptomyces parvulus* DPUA 1549, *Streptomyces owasiensis* DPUA 1748	cell-free extract	AgNPs	1–40	fcc	amino acids and peptides	antibacterial	[207]
*Streptomyces* sp. NH21	cell-free extract	AgNPs	ca. 44	fcc	proteins	antibacterial	[205]
	resting cells		ca. 8				
*Arthrobacter* sp. B4	EPS	AgNPs	9–72	fcc	EPS	antibacterial	[245]
*Micromonospora* KPMS10	cell-free extract	AgNPs	ca.80	N.D.	N.D.	antibacterial	[268]
*Streptomyces* sp. Al-Dhabi 89	cell-free extract	cubic AgNPs	4–11	fcc	extracellular metabolites	antibacterial	[211]
*Nocardiopsis alba*	cell-free extract	AgNPs	20–60	fcc	N.D.	antibacterial, antiviral	[269]
*Streptomyces spongiicola* AS-3	cell-free extract	AgNPs	ca.22	fcc	proteins	antibacterial	[220]
*Streptomyces* sp. 192ANMG and 17ANMG	cell-free extract	AgNPs	ca. 9 ca. 35	hcp ^4^	proteins	antibacterial, antibiofilm	[270]
**Fe-based NMs**							
*Actinobacter* strain EC5	growing cells	mixture of Fe_2_O_3_ and Fe_3_O_4_ NPs	10–40	fcc	extracellular biomolecules	superpara magnetic	[167]
*Actinobacter* sp.	growing cells	Fe_2_O_3_ NPs	5–7	fcc	proteins	superpara magnetic	[170]
		mixture of Fe_3_S_4_ and FeS_2_ NPs	ca. 20				
*Rhodococcus erythropolis* ATCC 4277	growing cells, cell-free extract	Fe_2_O_3_ NPs	50–100	fcc	N.D.	N.D.	[218,219]
**Cu-based NMs**							
*Streptomyces* sp. KUA106	cell-free extract	CuO NPs	100–150	N.D.	reductases	antibacterial, antifungal	[271]
	growing cells						
*Kocuria flava* M-7	cell-free extract	CuNPs	5–30	N.D.	proteins	N.D.	[195]
*Streptomyces capillispiralis* Ca-1	cell-free extract	CuNPs	5–59	fcc	proteins	antimicrobial, larvicidal	[213]
*Actinomycete* sp. VITBN4	cell-free extract	CuO NPs	ca. 60	N.D.	proteins	antimicrobial	[272]
*Streptomyces zaomyceticus* Oc-5; *Streptomyces pseudogriseolus* Acv-11	cell-free extract	CuO NPs	ca. 80	fcc	N.D.	antimicrobial, antioxidant, larvicidal	[216]
**Zn-based NMs**							
*Streptomyces* sp. KUA106	growing cells, cell-free extract	ZnO NPs	100–150	N.D.	reductases	antimicrobial	[271]
*Streptomyces* sp. *HBUM171191*	growing cells	ZnSO_4_ NPs	10–20	N.D.	N.D.	N.D.	[273]
*Streptomyces* sp.	growing cells	ZnO NPs	N.D.	N.D.	N.D.	antibacterial	[274]
*Rhodococcus pyridinivorans* NT2	growing cells, cell-free extract	ZnO NPs	ca. 100	N.D.	metabolites and proteins	UV-protective, photocatalytic, self-cleaning, antibacterial	[193]
**SeNMs**							
*Streptomyces bikiniensis* strain Ess_amA-I	growing cells	SeNPs	50–100	N.D.	proteins and enzymes	N.D.	[198]
		SeNRs ^5^	600			anticancer	
*Streptomyces minutiscleroticus* M10A62	resting cells	SeNPs	100–250	monoclinic	proteins	antibiofilm, antioxidant, wound healing, anticancer, antiviral	[198]
*Streptomyces* sp. ES2-5	growing cells	SeNPs	100–500	N.D.	N.D.	N.D.	[203]
*Rhodococcus etherivorans* BCP1	growing cells	SeNPs SeNRs	50–600	N.D.	amphiphilic biomolecules	N.D.	[208]
*Frankia inefficax* strain EuI1c	growing cells	SeNPs	N.D.	N.D.	N.D.	N.D.	[217]
*Streptomyces* sp. M10A65	resting cells	SeNPs	20–150	monoclinic	proteins	antibacterial, larvicidal, anthelminthic	[222]
**TeNMs**							
*Rhodococcus etherivorans* BCP1	growing cells	TeNRs	100–500	N.D.	amphiphilic biomolecules	N.D.	[204]
	resting cells	TeNPs TeNRs	200–700	trigonal		electrically conductive	[209]
**Co-based NMs**							
*Brevibacterium casei*	cell-free extract	Co_3_O_4_ NPs	ca. 6	crystals	proteins	magnetic	[172]
Mn-based NMs							
*Streptomyces* sp. *HBUM*	growing cells	MnSO_4_ NPs	10–20	N.D.	N.D.	N.D.	[273]

^1^ NPs indicates nanoparticles. ^2^ fcc stands for face-centered cubic. ^3^ N.D. indicates metal(loid) NM features not determined. ^4^ hcp stands for hexagonal close-packed. ^5^ NRs stands for nanorods
